# Prophylactic effect of compression stockings for elevated D‐dimer levels following endoscopic submucosal dissection

**DOI:** 10.1002/deo2.405

**Published:** 2024-07-15

**Authors:** Fumito Harada, Shinya Kodashima, Ken Ikusaka, Naoaki Aoki, Yuki Shimizu, Taku Honda, Miyoko Sakurai, Kyohei Maruyama, Hitoshi Aoyagi, Akari Isono, Ryo Miura, Koichiro Abe, Toshihiko Arizumi, Yoshinari Asaoka, Takatsugu Yamamoto, Atsushi Tanaka

**Affiliations:** ^1^ Department of Medicine Teikyo University School of Medicine Tokyo Japan; ^2^ Department of the Gastroenterology Ageo Central General Hospital Saitama Japan

**Keywords:** compression stockings, D‐dimer, deep‐vein thrombosis, endoscopic submucosal dissection, sedative

## Abstract

**Objectives:**

A relationship between endoscopic submucosal dissection (ESD) and deep vein thrombosis has been recognized. We previously reported that a high corrected midazolam dose (total midazolam dose/initial dose of midazolam used to induce sedation) is related to elevated D‐dimer levels after ESD. In this study, the effect of compression stockings (CSs) in preventing thrombosis following ESD under sedation was evaluated by measuring D‐dimer levels before and after ESD.

**Methods:**

The participants were patients who underwent ESD for upper gastrointestinal tumors during the period between April 2018 and October 2022. Patients with pre‐ESD D‐dimer levels ≥1.6 µg/m and patients with corrected midazolam doses ≤3.0 were excluded. A retrospective investigation of the relationship between CS use and high post‐ESD D‐dimer levels (difference in D‐dimer levels ≥1.0 µg/mL between before and after ESD) was conducted.

**Results:**

There were 27 patients in the non‐CS group (NCS) and 33 patients in the CS group. The number of patients with high post‐ESD D‐dimer levels was 13 (48.2%) in the non‐CS group and six (18.2%) in the CS group; the number in the CS group was significantly lower (*p* = 0.024). On logistic regression analysis, a relationship was seen between the wearing of CSs and a lower number of patients with high post‐ESD D‐dimer levels (odds ratio 0.24, 95% confidence interval 0.08–0.79, *p* = 0.019).

**Conclusion:**

Wearing CSs was related to a lower risk of high post‐ESD D‐dimer levels. This result suggests that thrombus formation is a cause of elevated D‐dimer levels after ESD.

## INTRODUCTION

In Japan, endoscopic submucosal dissection (ESD) has become one of the major treatments for gastrointestinal tumors with a very low likelihood of metastasis.^1^ Compared with surgical operations, there is a lower incidence of early complications, shorter hospital stays, and a higher overall survival rate with ESD.^2^, ^3^, ^4^ However, ESD procedures have a high level of difficulty and a high level of incidental risks such as perforation or bleeding.^5^, ^6^ The possibility of long procedure times has also been noted as a problem^5^, ^7^, ^8^; therefore, the use of sedatives is almost essential in endoscopic procedures such as ESD that take a long time, and maintaining a moderate to higher level of sedation during the procedure is considered desirable.^9^

It is well known that risk factors for deep vein thrombosis (DVT) include enhanced coagulability from surgical invasiveness, and being in the same position for a long period of time.^10^ The origin of the embolism in pulmonary thromboembolism, which could cause sudden death, is thought to be DVT in about 90% of cases,^10^ making the prevention and treatment of DVT crucial. Kusunoki et al. reported that asymptomatic DVT was seen in 10% of patients after ESD^11^ and it is thought that there may be a high risk of DVT in the post‐ESD period. However, there has been little discussion of specific risk factors associated with the occurrence of post‐ESD DVT.

We previously reported that D‐dimer levels are elevated in many patients after ESD for gastrointestinal tumors,^12^ and that the corrected midazolam dose (total midazolam dose/initial dose of midazolam used to induce sedation) is related to elevated D‐dimer levels after ESD.^13^ There are known to be individual differences in the sedative action of midazolam,^14^, ^15^, ^16^, ^17^ and the dose of midazolam needs to be carefully determined while considering patient age, general condition, concurrent medications, and other factors. The corrected midazolam dose is the dose corrected for individual differences with this drug, as well as a value that shows how many multiples of the midazolam dose used before the start of the endoscopic procedure are administered over the entire treatment period. It is assumed to be proportional to the time during which the moderate or higher level of sedation recommended in guidelines has been maintained.^9^ Therefore, the duration of a moderate or higher level of sedation is conjectured to be related to elevated D‐dimer levels.

D‐dimer is used as a marker that reflects increased secondary fibrinolysis, and it is often used in diagnosing conditions such as disseminated intravascular coagulation and DVT.^18^ Although D‐dimer levels are also known to be elevated in conditions other than a thrombotic tendency, such as inflammatory diseases, malignant tumors, cirrhosis, pregnancy, and aging,^10^ D‐dimer levels are widely used in clinical practice as an effective test to exclude disseminated intravascular coagulation and DVT^19^ since the existence of thrombi can be ruled out when D‐dimer levels are low. Investigations to date have not assessed the mechanisms that act in the elevation of D‐dimer levels after ESD. However, considering the relationship between the duration of moderate to higher levels of sedation and elevated D‐dimer levels in addition to reports that there is an increased risk of DVT occurring after ESD, it seems possible that elevated D‐dimer levels after ESD are due to a thrombotic tendency, and that there is a high possibility that this will cause DVT after ESD.^13^ Therefore, at our hospital it has been recommended since November 2020 that ESD patients with upper gastrointestinal tumors wear compression stockings (CSs) as a means of preventing DVT.

The effect of wearing CSs on preventing DVT after sedated ESD, based on measurements of D‐dimer before and after ESD, was assessed.

## SUBJECTS AND METHODS

### Subjects

The participants in this study were patients who underwent ESD for upper gastrointestinal tumors (esophageal, gastric tumors) using intravenous anesthesia at our hospital during the period from April 2018 to October 2022, and whose D‐dimer (Sysmex Corporation) levels were measured before and after ESD. Pre‐ESD D‐dimer levels were measured at the time of hospital admission for ESD, and post‐ESD D‐dimer levels were measured in the morning of the day after ESD. Patients with pre‐ESD D‐dimer levels ≥1.6 µg/mL, in whom the existence of thrombosis at the stage before ESD could not be ruled out, and patients with corrected midazolam doses ≤3.0, who are thought to have a low risk of elevated D‐dimer levels after ESD, were excluded. Patients who did not wear CSs, mainly those before October 2020, were included as a non‐CS (NCS) group and patients who wore CSs starting in November 2020 were included as a CS group. The above study plan was approved by the ethics committee of Teikyo University School of Medicine (approval no. 20‐141‐2).

### ESD technique

All ESD procedures were performed by or under the guidance of an endoscopy instructor with at least 5 years of experience performing ESD. Sedatives and analgesics were administered during ESD as described below while monitoring the patient's level of consciousness, respiration, and hemodynamics. Compression stockings are put on the patients when they leave the ward on the day of ESD and are worn continuously until it is confirmed in the days after treatment that there are no procedure‐related complications.

### Sedative and analgesic regimens at our hospital

Midazolam was used in all patients as a sedative. Midazolam 0.5–2 mg was intravenously administered before insertion of the endoscope, after which additional midazolam in amounts of 0.5–1 mg each was added as needed while monitoring the level of sedation. When the level of sedation at which treatment could be started was attained, the endoscope was inserted at a stage determined by the endoscopist (the dose up to this point was taken to be the initial dose of midazolam). When sedation began to wear off during the procedure and an additional sedative was judged necessary, additional midazolam in amounts of 0.5–1 mg each time was added at the discretion of the endoscopist. When sedation was not maintained even with the addition of midazolam, haloperidol was added together with the midazolam to maintain sedation.

For analgesia, either pentazocine hydrochloride or pethidine hydrochloride was used at the discretion of the endoscopist. Pentazocine hydrochloride 7.5 mg or pethidine hydrochloride 17.5 mg was administered intravenously as an initial dose. After the procedure was started, an additional analgesic was administered as needed at the discretion of the endoscopist.

After the entire procedure was completed and the endoscope was removed, 0.5 mg of the benzodiazepine antagonist Anexato was administered as needed and, upon confirming that the patient had awakened, the patient was moved out of the endoscopy unit. Patients rested in bed for 2 hours after the procedure. If there were no procedure‐related complications, they were then allowed to walk.

### Outcomes

The main outcome was to retrospectively demonstrate that wearing CSs suppresses increases in high D‐dimer levels after ESD.

### Statistical analysis

The statistical analysis was done using JMP 13.2.1 (SAS Institute Inc.). A difference in D‐dimer levels of ≥1.0 µg/mL between before and after ESD was taken to be a high post‐ESD D‐dimer level, and Fisher's exact test and Welch's *t*‐test were used to compare demographic factors between the CS and the NCS group. In addition, a logistic regression analysis was performed with whether or not CSs were used, corrected midazolam dose, and procedure duration as explanatory variables, and the relationship between high post‐ESD D‐dimer levels and whether or not CSs were used was examined. *p* < 0.05 was taken to indicate a significant difference.

## RESULTS

**FIGURE 1:**
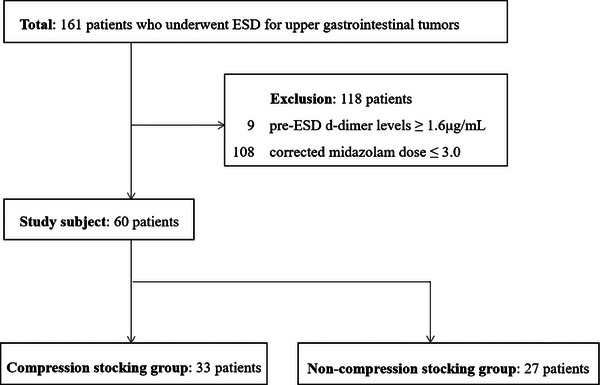
Flowchart of the study. ESD, endoscopic submucosal dissection.

In the period from April 2018 to October 2022, 161 patients underwent ESD for upper gastrointestinal tumors using sedatives at our hospital, and their D‐dimer levels were measured before and after ESD. Of the 161 patients in this study, 105 patients wore CSs, and 66 did not. After excluding patients with pre‐ESD D‐dimer levels ≥1.6 µg/mL (nine patients) and patients with a corrected midazolam dose ≤3.0 (109 patients), there were 33 patients in the CS group and 27 patients in the NCS group (Figure 1).

Table 1 compares the demographic factors between the CS group and the NCS group. The procedure duration was 202.6 ± 67.6 and 150.6 ± 49.8 min, respectively, significantly longer in the CS group (*p* = 0.002). The total midazolam dose was 8.0 ± 1.8 and 6.8 ± 2.6 mg, respectively, significantly higher in the CS group (*p* = 0.016), and the corrected midazolam dose was 4.4 ± 1.2 and 4.7 ± 1.1, respectively, which was not significantly different. The number of patients in whom pentazocine was used was 0 in the CS group and 10 in the NCS group, and the number of those in whom pethidine hydrochloride was used was 33 in the CS group and 18 in the NCS group. Both were significantly different (pentazocine use: *p* = 0.001, pethidine hydrochloride use: *p* = 0.001). The number of patients with high post‐ESD D‐dimer levels was six in the CS group and 13 in the NCS group; there were significantly fewer patients with high post‐ESD D‐dimer levels in the CS group (*p* = 0.024). None of the patients in both groups had clinically symptomatic DVT.

**TABLE 1:** Comparison of demographic factors between the compression stocking group and the non‐compression stocking group.

	Compression stocking group (*n* = 33)	Non‐compression stocking group (*n* = 27)	*p*‐value
**Sex (male/female)**	27/6	18/9	0.178
**Age (years)**	70.8 ± 8.71	74.4 ± 9.76	0.066
**ESD site (esophagus/stomach)**	10/23	9/18	0.801
**Thrombosis (*n*)**	5	5	0.728
**Hypertension (*n*)**	20	15	0.693
**Hyperlipidemia (*n*)**	7	8	0.454
**Diabetes mellitus (*n*)**	11	7	0.387
**Dialysis (*n*)**	0	1	0.265
**Antiplatelet drug use (*n*)**	7	3	0.296
**Anticoagulant use (*n*)**	3	2	0.814
**Procedure duration (min)**	202.6 ± 67.6	150.6 ± 49.8	0.002^a^
**Total midazolam dose (mg)**	8.0 ± 1.8	6.8 ± 2.6	0.016^a^
**Corrected midazolam dose**	4.4 ± 1.2	4.7 ± 1.1	0.104
**Pentazocine hydrochloride use (*n*)**	0	10	0.001^a^
**Total pentazocine hydrochloride dose (mg)**	‐	15.6 ± 7.1	‐
**Pethidine hydrochloride use (*n*)**	33	18	0.001^a^
**Total pethidine hydrochloride dose (mg)**	56.2 ± 20.4	54.4 ± 23.9	0.941
**Haloperidol use (*n*)**	21	13	0.271
**Total haloperidol dose (mg)**	5.0 ± 1.9	5.4 ± 2.5	0.722
**High post‐ESD D‐dimer levels (*n*)**	6(18.2%)	13(48.2%)	0.024^a^
**Symptomatic DVT (*n*)**	0	0	‐

Continuous value variables are described as mean ± standard deviation. ESD, Endoscopic submucosal dissection.

^a^ : *p* < 0.05.

Table 2 shows the results of logistic regression analysis with wearing/not wearing CSs and corrected midazolam dose as explanatory variables. Wearing CSs was the only variable related to fewer patients with high post‐ESD D‐dimer levels (odds ratio 0.24, 95% confidence interval 0.08–0.79, *p* = 0.019).

**TABLE 2:** Results of logistic regression analysis.

	Change in D‐dimer from before to after ESD					
	<1.0 (*n* = 41)	≥1.0 (*n* = 19)	Univariate	OR	95% CI	Multivariate
**Wearing of compression stockings (*n*)**	27	6	0.013^a^	0.24	0.08–0.79	0.019^a^
**Procedure duration (min)**	179.9 ± 66.5	177.7 ± 64.0	0.949			
**Total midazolam dose (mg)**	7.67 ± 2.34	6.89 ± 2.19	0.293			
**Corrected midazolam dose**	4.45 ± 1.19	4.67 ± 1.11	0.267	0.91	0.55–1.50	0.728
**Total pethidine hydrochloride dose (mg)**	55.2 ± 18.4	59.3 ± 23.4	0.554			
**Total haloperidol dose (mg)**	5.13 ± 2.06	5.18 ± 2.29	0.917			
**Thrombosis (*n*)**	8	2	0.385			
**Hypertension (*n*)**	25	10	0.542			
**Hyperlipidemia (*n*)**	10	5	0.873			
**Diabetes mellitus (*n*)**	13	5	0.672			
**Dialysis (*n*)**	0	1	0.139			
**Antiplatelet drug use (*n*)**	7	3	0.901			
**Anticoagulant use (*n*)**	5	0	0.112			

^a^ : *p* < 0.05.

## DISCUSSION

Wearing CSs is one of the major means of DVT prophylaxis.^10^, ^20^ Compared with other DVT prophylactic methods, it has fewer complications and is simple and inexpensive. Thus, it is a widely used method. In this study, the use of CSs by patients undergoing ESD for upper gastrointestinal tumors was shown to be related to a lower risk of elevated D‐dimer levels after ESD.

In the present study, high post‐ESD D‐dimer levels were assessed with the difference in D‐dimer levels between before and after ESD set to ≥1.0 µg/mL. The upper limit for a normal D‐dimer level is 1.0 µg/mL, and there are reports that, in patients prior to surgery, DVT can be ruled out with a D‐dimer cutoff value of 1.5 µg/mL.^21^ On the other hand, the cutoff value for the postoperative D‐dimer level, which is related to the occurrence of DVT after ESD, is 1.9 µg/mL,^11^ and a difference of 1.0 µg/mL in D‐dimer between before and after ESD is thought to significantly increase the possibility that DVT will occur due to a thrombotic tendency after ESD.

The corrected midazolam dose is the total dose of midazolam divided by the initial dose. It is a value corrected for the individual differences in the sedative effect of midazolam.^13^ We previously reported that when the corrected midazolam dose increases, the risk of elevated D‐dimer after ESD also increases.^13^ In the present study, with the purpose of focusing only on a high‐risk group for elevated D‐dimer levels after ESD, patients with a corrected midazolam dose ≤3.0 were excluded since our previous study found that only 6.1% of cases with an elevated D‐dimer were present in cases with corrected midazolam dose ≤3.0 and that the number of cases with elevated D‐dimer increased to 41% when corrected midazolam dose was greater than 3.0. In this investigation of a high‐risk group for elevated D‐dimer levels after ESD, high post‐ESD D‐dimer levels were seen in 48.2% of patients in the NCS group, with significantly fewer (18.2%) in the group that wore CSs as DVT prophylaxis. Compression stockings were judged to have an inhibitory effect on the elevation of D‐dimer levels after ESD.

To investigate whether new thrombus generation is inhibited by preventing the occurrence of DVT with CSs in the present study, it was necessary to exclude patients with a possible thrombotic tendency prior to ESD. Kawaguchi et al. reported that preoperative DVT could be ruled out by taking a preoperative D‐dimer level of 1.5 µg/mL as a cutoff value in gynecological diseases.^20^ In the present study, by excluding patients with pre‐ESD D‐dimer levels ≥1.6 µg/mL, the investigation was conducted with patients limited to those in whom DVT was assumed to not be present before ESD. In previous studies, an elevated D‐dimer level was seen in patients with a high corrected midazolam level, that is, patients in whom the sedative effect was continued for a long time. From this, it was conjectured that an elevated D‐dimer level is caused by a postoperative thrombotic tendency from being in the same position for a long period. However, there are no reports of how frequently DVT occurs in patients with elevated D‐dimer levels, and in our past studies as well, the presence or absence of DVT was not evaluated in all patients. Thus, it has not been demonstrated that patients with elevated D‐dimer levels show a thrombotic tendency. As a result of having patients who were not thought to have a thrombotic tendency prior to ESD wear CSs for DVT prophylaxis in the present study, the elevation of D‐dimer levels after ESD was significantly inhibited compared with patients in whom DVT prophylaxis was not carried out. Therefore, the hypothesis that the cause of elevated D‐dimer levels after ESD is a thrombotic tendency is correct. The thrombotic tendency was inhibited by wearing CSs, and a prophylactic effect on DVT, which is reported to be seen in about 10% of patients after ESD,^11^ was thought to have been obtained.

Since patients with a corrected midazolam dose ≤3.0 were excluded from the present study, it may be thought that ESD patients in whom the endoscopic procedure was completed in a short time were excluded. In fact, the procedure duration in the CS and NCS groups was 202.6 ± 67.6 and 150.6 ± 49.8 min, respectively, which was long in both cases. The procedure duration tended to be significantly longer in the CS group, but since the CS group consisted of patients in November 2020 or later, the cause is thought to be that many of the endoscopists had changed and that the endoscopists’ skill in performing ESD differed in the two groups. In this investigation, in addition to the longer procedure duration in the CS group, a tendency for significantly larger total doses of midazolam was seen. Even so, there was no significant difference in the corrected midazolam dose, for which a relationship with postoperative elevated D‐dimer levels has been suggested. However, it is also thought that a difference in endoscopist background in the two groups may have affected not only procedure duration but also how midazolam was used in each group. A logistic regression analysis was conducted with wearing/not wearing CSs, corrected midazolam dose, and procedure duration as explanatory variables, and this multivariate analysis showed that only the variable of whether or not CSs were worn was related to a decrease in the number of patients with a difference in D‐dimer levels ≥1.0 µg/mL between before and after ESD.

In endoscopic treatment, the use of both a sedative and an analgesic enhances the sedative effect; the possibility that the dose of sedative can be reduced has also been suggested.^9^ In our hospital, pentazocine or pethidine hydrochloride is used as an analgesic, but which to use is left to the discretion of the endoscopist. As mentioned above, the background of endoscopists in the two groups differed in this study, and as a result the tendencies in selecting a sedative also differed. In a past report, the amount and type of sedative were not shown to be related to elevated D‐dimer levels after ESD,^13^ but the number of patients in that study was small, and that assessment was insufficient. In the present study as well, since pethidine hydrochloride was used in all patients in the CS group, its influence cannot be sufficiently evaluated, and this remains a matter for further investigation.

Considering the results of the present study, DVT prophylaxis is thought to be desirable for patients whose corrected midazolam dose is high; that is, patients whose endoscopic treatment may require a long time under sedation. Since CSs are a DVT prophylactic method that is inexpensive and for which there are few contraindicated diseases,^10^, ^21^ it is thought that DVT prophylaxis with CSs should be considered for endoscopic procedures performed under sedation.

This was a retrospective study with a small number of patients since only high‐risk patients were included. This and the slight differences in background between the two groups are limitations, and further investigation is considered necessary.

## CONFLICT OF INTEREST STATEMENT

None.

## ETHICS STATEMENT

This study was approved by the ethics committee of Teikyo University School of Medicine (approval no. 20‐141‐2).
